# The changing relationship between racial identity and skin color in Brazil

**DOI:** 10.1073/pnas.2411495121

**Published:** 2024-12-30

**Authors:** Nicholas C. Freeman, Edward E. Telles, Rachel E. Goldberg

**Affiliations:** ^a^Department of Sociology, University of California, Irvine, CA 92697-5100

**Keywords:** race, identity, skin color, racial boundaries, Brazil

## Abstract

In recent years, Brazil’s non-White (Brown and Black) population became a numerical majority for the first time since the 19th century. Although we know this change was mostly due to racial reclassification, we do not know how such changes are related to skin color, the primary marker of race in Brazil. Using data from six Latin American Public Opinion Project (LAPOP), or America’s Barometer, surveys from 2010 to 2023, we examine how changes in racial self-identification (White, Brown, or Black) are related to respondent skin color (light, medium, or dark). We ask how the association between self-identified race and skin color changed over the 13-y period and to what extent these trends varied by educational level. We found a “darkening” of racial identification over time, especially among those with medium and dark skin, who increasingly identified as Black. Brazilians with light skin increasingly identified as Brown, but this was partly explained by changes in interviewer reporting of skin color in the same period. We found only minor evidence that education level was related to changing racial classification over time. Our findings further understanding of recent trends in racial reclassification, which may reflect growing Black consciousness and the extension of racial quotas to both Brown and Black Brazilians in federal universities and public sector jobs.

## Significance

Brazil’s racial composition has changed dramatically in recent years, mostly because of changing racial identities. However, even though race is based on physical characteristics like skin color, we know little about how the two are related empirically. This study investigates the changing relationship between Brazil’s racial composition and skin color. Specifically, we examine changes in the relationship between Brazilian racial self-identification and interviewer-rated skin color from 2010 to 2023, a period when robust racial quotas were extended throughout Brazil. We generally find a “darkening” of racial identification among persons of all skin colors, despite alternate theoretical expectations. We discuss unique patterns in racial identity, consciousness, and boundary-crossing and their implications for Brazil’s racial composition and inequality.

Brazil is home to the largest African-origin population outside of Africa. Moreover, according to Brazilian Censuses and official household surveys, Brazil recently became a majority non-White country for the first time since about 1900 (1–3). From 2000 to 2022, Brazil’s self-identified White (*branco*) population declined from 54 to 45% of the national population while the Black (*preto* or *negro*) population nearly doubled, from 6 to 11%, and the Brown (*pardo*) population increased from 39 to 43% (2, 4). One study shows that the growth of Brazil’s Brown and Black population from 1990 to 2010 is primarily because of racial reclassification rather than demographic change (5). The recent darkening of Brazil’s population contrasts with changing racial composition in most of the 20th century, when the White population grew primarily because of immigration from Europe and, to a lesser extent, reclassification in the direction of whitening (3, 6).

Racial classification in Brazil is highly fluid and depends primarily on one’s appearance, particularly skin color (7–10). However, the Brazilian Census and official surveys classify respondent race based on self-identification, as is international practice. Moreover, they ask respondents to self-identify based on their “race or color,” (3, 7) although scholars of race have emphasized analytical differences between racial self-identity and perceived skin color (8, 11). The country’s relatively fluid racial boundaries allow Brazilians to self-identify in categories that do not correspond with their color or how they are perceived by others (12–14), and their racial self-identification may change over time (15). Despite the importance of skin color to defining a person’s race, we do not know whether or how changes in Brazil’s official racial composition, and more specifically self-identification, are related to skin color.

This analysis examines how changes in Brazil’s racial composition are related to skin color. Thus, we disentangle two central dimensions of race: self-identified race and perceived race in the form of interviewer-rated skin color (12). We benefit from a unique, biannual survey of Brazil, which began collecting interviewer-rated skin color data in 2010. Specifically, we examine changes in the relationship between racial self-identification and skin color from 2010 to 2023. Among our findings, we identify a remarkable transvaluation of the racial hierarchy in which many Brazilians have shifted their racial identities, net of skin color, to increasingly darker categories over this short period.

## Literature Review

Although both Brazil and the United States share a racial/color hierarchy with whiteness at the top and blackness at the bottom, racial identities contrast sharply in the two countries. In particular, racial boundaries, particularly between persons of European and African descent, are relatively soft in Brazil, where individual racial identities are often fluid. Compared to the United States, race in Brazil is ambiguous and fluid, there is relatively little sense of racial groupness, there is no history of racial classification (e.g., one-drop) rules and political attitudes are rarely differentiated by race (2, 3, 8, 9, 14). Also, most non-White Brazilians have typically chosen to identify as mixed-race rather than as Black or White, revealing a racial continuum rather than a biracial system. However, that has begun to change, as some scholars have suggested Brazil is becoming more biracial and race-conscious while the United States is becoming more multiracial and fluid (9, 16).

Recent changes in Brazilian racial identity are likely the result of a growing Black consciousness as well as the emergence of racial policies that incentivize non-White identities (2, 17, 18). Although growing racial consciousness has origins at least since the 1970s, new racial policies in Brazil began in the early 2000s. Racial quotas in Brazil, geared toward expanding mobility opportunities for all Brazilians, may be the biggest and boldest racial policies in the Americas. Unlike affirmative action in the United States, racial quotas directly call for public universities and other public institutions to create a fixed number of positions to be filled by Black, Brown, and Indigenous persons as well as persons of lower-class origins (2, 19). The most well-known racial quotas policies began in 2003 at a small number of public universities and expanded in subsequent years (2, 3). By 2012, a Supreme Court decision affirmed the constitutionality of these policies and later that year, the Brazilian Congress mandated racial quotas for all federal universities (2, 20). In 2014, the Brazilian Congress further extended racial quotas to all new applications for civil service jobs. Moreover, responding to accusations of “racial fraud,” the 2012 and 2014 laws required the establishment of racial verification commissions to monitor whether a candidate’s self-identification matched their actual appearance (20).

Concurrently, scholars have found that a status- or money-whitening effect that predominated in Brazil in much of the 20th century (7, 10, 21) shifted to a “status darkening” effect in recent years (2, 11, 17). “Money whitening” refers to how upward social mobility can lighten one’s racial classification or identity, which has also been observed in the United States (22). Status- or “money-darkening” refers to the opposite phenomenon of more recent years. This apparent reversal seems to reflect changing incentive structures in the Brazilian context, where racial boundaries are fluid. Whereas past Brazilian ideas of race (which considered socioeconomic status) and racial hierarchies incentivized dark persons with high status to identify in lighter categories (“whiten”) (7, 21), racial quotas now create incentives to identify in darker categories (2, 17). In particular, Brazil’s new system of racial quotas is aimed at higher education and civil service jobs, which are selective of persons with more education. For example, scholars have found university applicants to reclassify themselves from White to non-White in response to racial quotas and the valorization of Black identity and culture (17, 18, 23).

Changing racial classification or identification is often theorized in the language of racial/ethnic boundaries, where changes may be due to boundary crossing, boundary shifting, or boundary blurring (24, 25). Loveman and Muniz (24), for example, found that the racial boundary shifted in Puerto Rico between 1910 and 1920. In particular, they found that Puerto Ricans increasingly identified as White. They assumed, but could not substantiate, that the White category expanded only because mixed-race persons increasingly classified as White. They found no support for boundary crossing, which they defined as changing racial identity in response to the acquisition of a new trait such as more education (21, 22). Overall, despite their important conceptual and empirical breakthrough, they were unable to empirically examine these changes in relation to phenotype or skin color.

The context in Brazil today has reversed, toward the apparent contraction rather than expansion of whiteness. We build on Loveman and Muniz’s (24) work by empirically examining Brazil’s shifts in racial self-identification in conjunction with skin color. In both cases, the White–non-White boundary shifted; whiteness expanded in Puerto Rico a century ago but nonwhiteness has apparently expanded in the current Brazilian case. Moreover, we also examine changes in the Brown–Black boundary. This study examines racial identity changes in relation to skin color, including their response to institutional changes and the politicization of Black identities.

### Hypotheses.

Given that research has shown a growth of the non-White population in Brazil, we hypothesize five potential drivers of this trend in relation to skin color

H1. Darkening among persons of all skin colors.

H2. Status effects, where upward mobility and incentives from racial quota policies may increasingly lead persons, especially those with more education, to identify in non-White categories (11, 18, 23).

H3. Increasing alignment between skin color and racial self-identification across the racial spectrum, where light skin persons increasingly identify as White, medium skin persons as Brown, and dark skin persons as Black. This would be consistent with non-White expansion because dark and medium skin color persons may have been previously less likely to self-identify in Black and Brown categories than light skin persons were to self-identify as White. This also addresses the persistence/convergence of distinctions between racial identity and skin color (8, 17)

H4. Growth in non-White identities among only medium and dark skin persons stemming from Brazilian institutional changes. Despite growing incentives to darken racial identities among persons across the color spectrum, racial verification may have led light skin Brazilians to restrict their identification in non-White categories (20).

H5. Expansion primarily into the Black category. The politicization and valorization of Black identities, including a recent discourse that Blacks are more deserving than mixed-race persons of racial quotas (2, 20), may lead some to increasingly identify as Black rather than Brown.

## Results

### Descriptive Findings.

Table 1 depicts the distributions of racial self-identification and skin color for each survey year and in the pooled sample. Overall, 47.6% of respondents in the analytic sample pooled across years self-identified as Brown, followed by 36.1% who identified as White, and 16.2% who self-classified as Black. This distribution closely mirrors how interviewers rated skin color, with 46.3% of respondents rated as medium skin tone, 39.3% as light, and 14.2% as dark. *SI Appendix*, Fig. S1 further depicts the relationship between racial self-identification and skin color on the 11-point scale for the pooled sample. In particular, it reveals a tendency for light skin persons (1 to 3) to identify as White, medium skin persons (4 to 6) as Brown, and dark skin persons (7 to 11) as Black, but with some overlap between categories.

**Table 1.** Respondent racial self-identification and interviewer-rated respondent skin color, distribution by survey wave

	2010	2012	2014	2016/17	2019	2023	Pooled
Racial self-identification							
White	39.2	38.1	37.7	34.6	32.0	32.4	36.1
Brown	50.1	46.5	49.2	45.7	45.2	47.0	47.6
Black	10.6	15.3	12.9	19.6	22.7	20.5	16.2
Respondent skin color (3-category)							
Light	35.8	33.7	39.4	44.6	42.1	43.2	39.3
Medium	50.2	45.9	45.4	43.7	45.8	44.2	46.3
Dark	13.8	20.3	15.0	11.5	11.9	12.5	14.2
Respondent skin color (11-point scale)							
Mean respondent skin color	4.41	4.71	4.28	4.06	4.21	4.12	4.32
SD	2.04	2.43	2.10	1.92	2.02	2.13	2.12
*N*	2256	1407	1326	1270	1237	1308	8804

Percentages are presented unless otherwise noted. Source: Latin American Public Opinion Project (LAPOP), six survey rounds: 2010, 2012, 2014, 2016/17, 2019, and 2023.

Table 1 also reveals shifts in the distributions of racial self-identification and, to a lesser extent, in interviewer-rated skin color across survey years. The most notable change in self-identification is a near-doubling of identification as Black across the period (10.6% of the sample in 2010 compared to 20.5% of the sample in 2023). By contrast, interviewers generally placed more respondents into the lighter end of the skin color scale over time, although the increase was less dramatic than with racial identity (35.8% of the sample were categorized as “light” in 2010 compared to 43.2% in 2023). *SI Appendix*, Table S1 provides descriptive statistics for the other independent variables of interest.

### Regression Results.

In Table 2, we predict racial self-identification as Brown or Black (reference = White) using multinomial logistic regression analysis. Model 1 includes interviewer-rated skin color and the controls; Model 2 adds interactions between skin color and survey year.

**Table 2.** Multinomial regressions predicting Brown and Black versus White racial identity

	Model 1 (without interactions)		Model 2 (with interactions)	
	Brown	Black	Brown	Black
	*b*	*b*	*b*	*b*
Respondent skin color (reference = light)				
Medium	2.439*** (0.068)	3.896*** (0.154)	2.424*** (0.128)	3.324*** (0.317)
Dark	4.144*** (0.238)	7.777*** (0.275)	4.302*** (0.371)	7.710*** (0.460)
Year	0.138*** (0.016)	0.348*** (0.022)	0.137*** (0.021)	0.249*** (0.059)
Female	0.124* (0.063)	0.184* (0.091)	0.125* (0.063)	0.181* (0.091)
Age	−0.014*** (0.002)	−0.018*** (0.003)	−0.014*** (0.002)	−0.019*** (0.003)
Education (reference = primary)				
Secondary	0.020 (0.078)	0.196 (0.110)	0.021 (0.078)	0.194 (0.110)
College	−0.319** (0.107)	−0.115 (0.156)	−0.318** (0.107)	−0.122 (0.157)
Wealth	−0.075* (0.035)	0.008 (0.050)	−0.075* (0.035)	0.011 (0.050)
Urban	0.334*** (0.097)	0.496*** (0.154)	0.334*** (0.097)	0.489*** (0.153)
Region (reference = southeast)				
North	1.120*** (0.107)	0.154 (0.164)	1.119*** (0.107)	0.145 (0.165)
Northeast	0.989*** (0.092)	0.947*** (0.124)	0.988*** (0.092)	0.942*** (0.124)
Central-west	0.677*** (0.100)	−0.002 (0.156)	0.676*** (0.100)	−0.001 (0.156)
South	−1.236*** (0.087)	−0.823*** (0.128)	−1.237*** (0.087)	−0.821*** (0.129)
Interviewer skin color (reference = light)				
Medium	−0.103 (0.066)	−0.190* (0.094)	−0.102 (0.066)	−0.192* (0.095)
Dark	−0.033 (0.147)	0.070 (0.191)	−0.035 (0.147)	0.089 (0.190)
Respondent skin color × year interaction				
Medium × year			0.006 (0.033)	0.149* (0.067)
Dark × year			−0.069 (0.112)	−0.047 (0.122)
Pseudo R^2^	0.319		0.319	

Pooled sample, *N* = 8,804. Beta coefficients are presented. Robust SE in parentheses. Source: LAPOP, six survey rounds merged, 2010, 2012, 2014, 2016/17, 2019, and 2023. *P < 0.05, **P < 0.01, ***P < 0.001 (two-tailed tests).

**Fig. 1.**
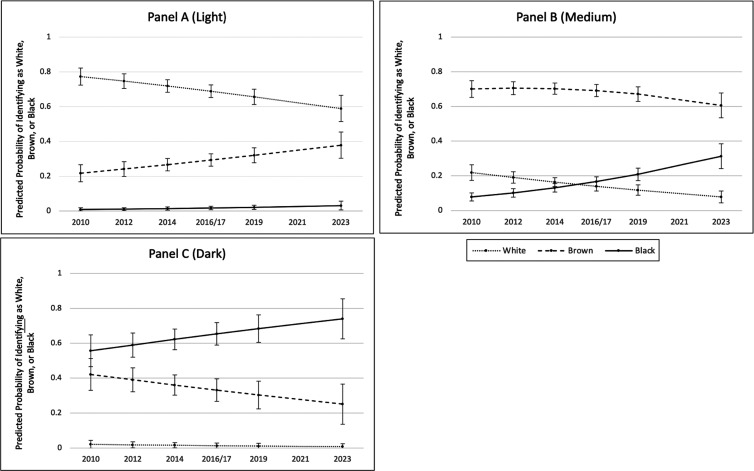
Predicted probabilities of self-identity as White, Brown or Black for light skin persons (panel *A*), medium skin persons (panel *B*), and dark skin persons (panel *C*).

The skin color–year interactions in Model 2 are best interpreted with predicted probabilities, which we show in Fig. 1. We present a panel for each of the skin color groups, with separate lines for identification as White, Brown, and Black over time. Fig. 1*A* shows that the predicted probability that a light skin person identifies as Brown increased substantially over the 13-y period. In particular, the predicted share of light skin persons identifying as Brown almost doubled from 22 to 38%, even though a (declining) majority continued to identify as White. Fully 77% of light skin persons were predicted to self-identify as White in 2010, with a decline to 59% in 2023. Rarely did a light skin person identify as Black in any wave.

Panel *B* shows that most medium skin persons continued to identify as Brown across time, though White identification decreased and Black identification markedly increased. Predicted self-identification as White decreased from 23% in 2010 to under 7% by 2023. The largest shift among medium skin persons was in Black identity. Predicted self-identification as Black among medium skin color persons nearly quadrupled, from 8% in 2010 to 31% in 2023.

Panel *C* shows that many dark skin Brazilians also shifted their racial self-identification from Brown to Black in the 2010–2023 period. Just over half (56%) of dark skin persons were predicted to self-identify as Black in 2010; by 2023, this had increased to almost three-quarters (74%). Correspondingly, 42% of dark skin persons were predicted to identify as Brown in 2010 with a decrease to one-fourth (25%) in 2023. Very few dark skin persons identified as White in any survey year.

Overall, Fig. 1 results show that for both medium and dark skin persons, the biggest shifts in racial self-identification were toward the Black category. Thus, we find that Brazilians who can plausibly identify as Black increasingly identify as Black and eschew, to some extent, the Brown category. On the other hand, light skin color persons almost never claim the Black category but increasingly identify as Brown.

**Fig. 2.**
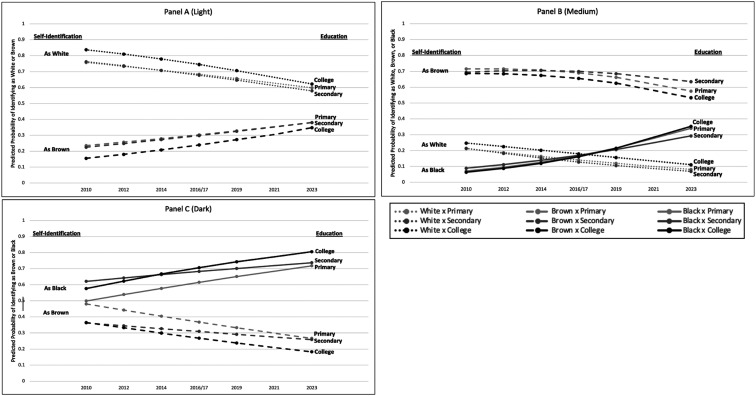
Predicted probabilities of self-identity as White, Brown, or Black, by education level, for light skin persons (panel *A*), medium skin persons (panel *B*), and dark skin persons (panel *C*).

To examine potential status effects, we incorporated three-way interactions of skin color, education, and survey year into our multinomial logistic regression models. Table 3 displays only the main and interaction effect results for these three variables. The other control variables shown in Table 2 are included in the Table 3 regression models but are not presented. Since the interaction results are cumulative across levels and thus difficult to interpret, we present predicted probabilities summarizing our results in Fig. 2.

**Table 3.** Multinomial regressions predicting Brown and Black versus White racial identity (three-way interaction with respondent skin color, education, and year)

	Brown		Black	
	*b*	(SE)	*b*	(SE)
Respondent skin color (reference = light)				
Medium	2.373***	(0.198)	3.313***	(0.662)
Dark	4.513***	(0.538)	7.950***	(0.815)
Year	0.118**	(0.038)	0.202	(0.134)
Education (reference = primary)				
Secondary	−0.066	(0.205)	0.463	(0.732)
College	−0.583*	(0.270)	−0.245	(0.976)
Respondent skin color × year interaction				
Medium × year	0.005	(0.056)	0.220	(0.143)
Dark × year	−0.156	(0.161)	−0.082	(0.204)
Education × year interaction				
Secondary	0.014	(0.049)	0.036	(0.152)
College	0.065	(0.061)	0.082	(0.198)
Respondent skin color × education interaction				
Medium × Secondary	−0.026	(0.276)	−0.203	(0.773)
Medium × College	0.423	(0.389)	0.001	(1.064)
Dark × Secondary	−0.259	(0.817)	−0.216	(1.068)
Dark × College	−0.992	(0.978)	−0.924	(1.319)
Respondent skin color × education × year interaction				
Medium × Secondary × year	0.035	(0.075)	−0.072	(0.167)
Medium × College × year	−0.099	(0.098)	−0.088	(0.220)
Dark × Secondary × year	0.223	(0.246)	0.127	(0.282)
Dark × College × year	0.148	(0.246)	0.143	(0.297)
Pseudo R^2^	0.320			

Pooled sample, *N* = 8,804. Beta coefficients are presented. Robust SE in parentheses. Controls included, but not presented. Source: LAPOP, six survey rounds merged, 2010, 2012, 2014, 2016/17, 2019, and 2023. *P < 0.05, **P < 0.01, ***P < 0.001 (two-tailed tests).

Fig. 2 shows how racial identification changes from 2010 to 2023 by level of education, for each of the three skin color groups (in separate panels). CI are not shown because they almost always overlapped and, given the large number of lines, obscured the main results.

Generally, Fig. 2 and Table 3 results suggest only minor educational heterogeneity in the patterns observed in Fig. 1. Among light skin persons (Panel *A*), self-classification as White decreased and self-identification as Brown increased similarly for all educational groups. Among medium skin color persons (Panel *B*), by 2023 those with secondary education were most likely to identify as Brown and those with college education least likely, whereas educational differences were minimal in 2010. Panels *B* and *C* show that identification as Black increased more steeply over time among medium and dark skin color persons with college education compared to those with secondary education, although these differences were not statistically significant (Table 3).

### Robustness Check.

It is possible that some of the observed darkening of racial self-identification over time could be an artifact of the tendency of interviewers to increasingly classify respondents on the light end of the skin color scale (Table 1). To test the robustness of our results to this explanation, we ran simulation multinomial logistic regressions (*SI Appendix*, Tables S2 and S3) and predicted probabilities (*SI Appendix*, Figs. S2 and S3) with a dataset in which we standardized the skin color distribution across survey years to match the 2023 distribution.

When the skin color distribution was kept constant across survey years, the changes in racial self-identification for those with light skin were less marked. *SI Appendix*, Fig. S2*A* shows that compared to Fig. 1*A*, the predicted probability that a light skin color person identifies as Brown increased to a lesser extent over the period. In particular, the predicted share of light skin persons identifying as Brown rose from only 30 to 35%. Thus, the lightening of interviewer-rated skin color over time likely accounted for some of the apparent increase in self-identification as Brown among those classified as having light skin.

By contrast, keeping the skin color distribution constant across survey years had virtually no bearing on the trends in racial self-identification over time for medium and dark skin persons. *SI Appendix*, Fig. S2*B* is nearly identical to its counterpart in Fig. 1, with predicted self-identification as Black among medium skin persons nearly quadrupling from 8% in 2010 to 31% in 2023. Similarly, differences between *SI Appendix*, Fig. S2*C* and Fig. 1*C* were negligible. Just over half (56%) of dark skin persons were predicted to self-identify as Black in 2010; by 2023, this had increased to almost three-quarters (72%). Overall, then, the observed increases in self-identification as Black among medium and dark skin persons were unlikely to have been driven by changes in interviewer-rated skin color.

## Summary and Discussion

In the past two decades, Brazil has become a majority non-White country, mostly because of changes in how Brazilians racially classify themselves. Because skin color is known to be the primary marker of race in Brazil, it is important to systematically examine the relationship between changing Brazilian racial identity and skin color.

We found mixed support for Hypothesis 1, with clear evidence of darkening among medium and dark skin Brazilians but a less robust trend for light skin Brazilians. In particular, medium skin persons tripled the extent to which they identified as Black over the 13-y period. Moreover, over three-quarters of dark skin persons identified as Black in 2023, compared to less than half in 2010. Our observed data for light skin persons suggested that they doubled their identification as Brown over time. However, our simulation accounting for changes in interviewer reporting of skin color suggested that much of this apparent trend stemmed from changes in how interviewers evaluate respondents on the light end of the skin color scale.

We found minimal evidence of status effects (money whitening or darkening) over time, providing only marginal support for Hypothesis 2. The overall shifts that we identified tended to be similar for persons at all educational levels. The most notable status effects we observed were that college-educated persons of medium- and dark-skin color became especially likely to identify as Black (compared to less educated counterparts) by 2023; however, these educational differences were marginal at best. This suggests that racial boundary crossing from status shifting may have diminished in Brazil compared to earlier periods, whether in the direction of whitening (7, 10, 21) or darkening (5, 11).

We observed growing alignment between racial identity and color only among dark skin persons, leading us to reject Hypothesis 3. We found no evidence that light skin persons increasingly identified as White over time, nor that medium skin persons increasingly identified as Brown. This reveals a changing relationship between racial identity and skin color, which is notable considering that racial identity on the Brazilian Census is based on race or color.

We found some support for Hypothesis 4 regarding the likely influence of verification commissions on the racial identity of light skin persons. The observed data showed that light skin persons increasingly self-identified as Brown over time despite racial verification. However, the simulation results provided more support for Hypothesis 4, indicating that once changes in interviewer classification of skin color were accounted for, the shifts in racial self-identification among light skin persons were minimal. The simulation suggests that light skin persons may in fact be responding to the university and civil-service monitoring of racial fraud, rather than to overall societal incentives to darken.

We observed sizable shifts of both medium- and dark skin persons into the Black category, supporting Hypothesis 5. We thus find a shift to blackness among persons that likely were traditionally seen as Brown (medium skin persons) as well as persons that were probably seen as Black (dark skin persons) but did not identify that way. That movement was likely triggered by an ongoing Black consciousness movement that values identity as Black (*negro* or, increasingly *preto*). We do not expect that the verification commissions had much role in Black identity since they monitor identity along the White–non-White boundary.

In sum, we identify that many Brazilians have shifted their racial identities to increasingly darker categories over this short period. This represents a historic change, suggesting a reversal in the valuation of Black identities in Brazil. Specifically, we find an expansion in the social definition of blackness from 2010 to 2023. In other words, we find a boundary shift to darkening at the middle and dark ends of the color continuum. This trend extended less robustly to light skin persons, perhaps because of growing identity policing. Consistent with these results, increasing alignment between color and status only occurred for dark skin persons. Finally, we did not find boundary crossing (money whitening or money darkening) from educational status.

These changes have major implications for the study of racial inequality. Since racial self-identification is the standard measure of inequality, shifts driven by racial reclassification of persons of the same skin color underscore that quantifications of racial inequality are in part artifacts of how race is measured (12, 15). For example, scholars have found that racial inequality in Brazil by skin color is greater than inequality based on racial identity (8, 26). Studies of racial disparities in other domains, such as in health, income, and criminal justice, should also consider how racial inequality might vary depending on the focal measure of race. Researchers documenting changing racial inequalities in Brazil should consider the implications of standard measures based on self-identification and, when possible, consider other measures of race like those based on skin color.

## Materials and Methods

### Data.

To examine changes in the relationship between racial self-identification and skin color in Brazil over time, we used data from six of the seven biannual surveys from the 2010 to 2023 (2010, 2012, 2014, 2016/17, 2019, and 2023) Brazil surveys of the LAPOP, also known as the Americas Barometer. These surveys are nationally representative of the Brazilian population aged 16 y and older and include information on self-identified race using Census categories and interviewer-rated skin color. The LAPOP sample is a probability sample of the noninstitutionalized voting age adult population in Brazil. The sampling design has remained virtually the same since 2010, with stratified multistage cluster sampling based on municipality size, urbanity, and region. Data were collected from one respondent per household using face-to-face survey interviews. To reduce any sample imbalances, we used a weighted dataset. We excluded the 2021 LAPOP survey from our analyses because interviews were conducted by telephone during the COVID-19 pandemic and thus the interviewer-rated skin color item was omitted. Although it may appear that LAPOP skipped a year from 2014 to 2017, the 2014 survey was administered at the end of 2014 and the 2016/17 survey at the end of 2016 and beginning of 2017, representing an interwave interval of roughly 26 mo.

### Dependent Variable.

Our dependent variable is self-identification as Brown (*pardo*)^*^, Black (*preto* or *negro*), or White (*branco)*. Fully 98.5% of the Brazilian population identified in one of these three racial categories, according to the 2010 Brazilian Census. The dependent variable was drawn from the following question in the LAPOP survey: “Do you consider yourself a person that is white, indigenous, black, brown, yellow or other?” This question was asked consistently across all six surveys and closely mirrors the item in the 2010 Brazilian Census, which is “Your color or race is _white, _black, _yellow, _brown, _indigenous” (27).

There are several notable differences between the LAPOP survey question and the Brazilian Census. Most notably, the 2010 Brazilian Census refers to “color or race,” as it has since 1990,^†^ while the LAPOP survey does not use either term. Additionally, LAPOP instructs interviewers to classify persons as Black if they insist on identifying as *Afro-Brasileiro*, or Afro-Brazilian. Another important distinction is that while the Census asks racial self-identification of only one person in each household (who racially classifies other household members), the LAPOP surveys directly ask each respondent to racially self-identify themselves. Finally, the LAPOP and Census differ regarding the order of the categories, and LAPOP includes “other” in the question itself.

### Independent Variables.

Our main independent variables are skin color, year, and education. Skin color is based on interviewer ratings of the respondent’s facial color, using a skin color palette containing 11 distinct skin tones (1 = lightest, 11 = darkest) (*SI Appendix*, Fig. S4). The palette was not shown to respondents during interviews. The color palette remained the same across survey waves. For most of the analysis, we collapsed skin color into three categories: light (1 to 3), medium (4 to 6), and dark (7 to 11). *SI Appendix* results using the full 11-point skin color scale can be found in *SI Appendix*, Table S4.

Year is a categorical variable referring to the six survey waves, which we operationalized continuously in our regressions with 1 for 2010, 2 for 2012, 3 for 2014, 4 for 2016/17, 5 for 2019, and 7 for 2023. We coded the 2023 year as 7 instead of 6 to account for the omission of 2021 data from our analyses. Education consists of three levels of highest educational attainment: primary (some or completed elementary school), secondary (some or completed high school), and college (some or completed college).

We additionally included the following control variables that may also affect racial identification: wealth, sex, age, urban/rural residence, and region. As has become standard in development research, we used principal component analysis (PCA) to generate a relative wealth measure based on a weighted index of possession of eight household assets such as a refrigerator, a computer, and a washing machine (28, 29). We operationalized age continuously, and sex and urban/rural residence dichotomously. Region is a five-category variable based on Brazil’s five official regions. Finally, we controlled for the skin color of the interviewer, using a continuous variable from 1 to 11, to account for potential interviewer bias in rating respondent skin color (11, 30). Interviewer supervisors used the same 11-point scale that was used to rate respondents’ skin color.

### Analytic Strategy.

To examine associations between skin color and racial self-identification over time, we used multinomial logistic regression models predicting racial self-identification as Brown or Black, compared to White. After examining average associations between skin color (light [ref], medium, dark) and racial self-identification across survey waves, we added interaction terms between skin color and survey year to observe change in these associations over time. Finally, we investigated a three-way interaction between educational attainment, skin color, and survey year. We generated predicted probabilities to aid in interpretation of all interaction results.

As a robustness check, we also simulated how the regression results would have differed if the distribution of interviewer-reported skin color had remained stable over time. To do so, we standardized the distribution of the three-category skin color measure in each survey wave to the 2023 distribution. After identifying how many respondents would need to be moved between categories to match the 2023 distribution, we randomly selected respondents for reallocation based on their 11-point skin color score, selecting respondents with scores near the threshold for a given category with a higher probability than those further from the threshold. For example, when randomly selecting individuals to reallocate from the “medium” to “light” category, we drew ⅔ of these individuals from the “4” skin color score. We reran all of the regression models with the standardized skin color variable for comparison with the results from the observed data.

## Supplementary Material

Appendix 01 (PDF)

## Data Availability

All our empirical results use data from the Latin American Public Opinion Project (LAPOP). We are not able to make these data directly available due to LAPOP’s terms and conditions for data usage. However, the data are publicly accessible and can be downloaded via https://www.vanderbilt.edu/lapop/ (31). Additionally, our analysis code is available upon request or can be found here: https://github.com/ncfreema/pnas_racial_identity_and_skin_color_in_Brazil (32).

## Footnotes

^*^Pardo specifically refers to the color of a particular envelope in Brazil. It also translated as “mixed race,” which would include mixtures of Black, White, and Indigenous origins (7, 14).

^†^Prior to the 1990 Census, it asked respondents simply about their “color” (and not “race”).
