# Supplementary material for: The changing relationship between racial identity and skin color in Brazil
Source: Proc Natl Acad Sci U S A. 2024 Dec 30;122(1):e2411495121. doi: 10.1073/pnas.2411495121 (PMC11725908; doi:10.1073/pnas.2411495121)
Supplement: Supplementary file 1 — Appendix 01 (PDF) [file pnas.2411495121.sapp.pdf]

Supporting Information for
The Changing Relationship between Racial Identity and Skin Color in Brazil

Nicholas C. Freeman

Edward E. Telles

Rachel E. Goldberg

University of California, Irvine

Corresponding Author: Nicholas C. Freeman

Email: ncfreema@uci.edu

This PDF file includes:

Figures S1 to S4
Tables S1 to S4

Figure S1 Self-identification by skin color

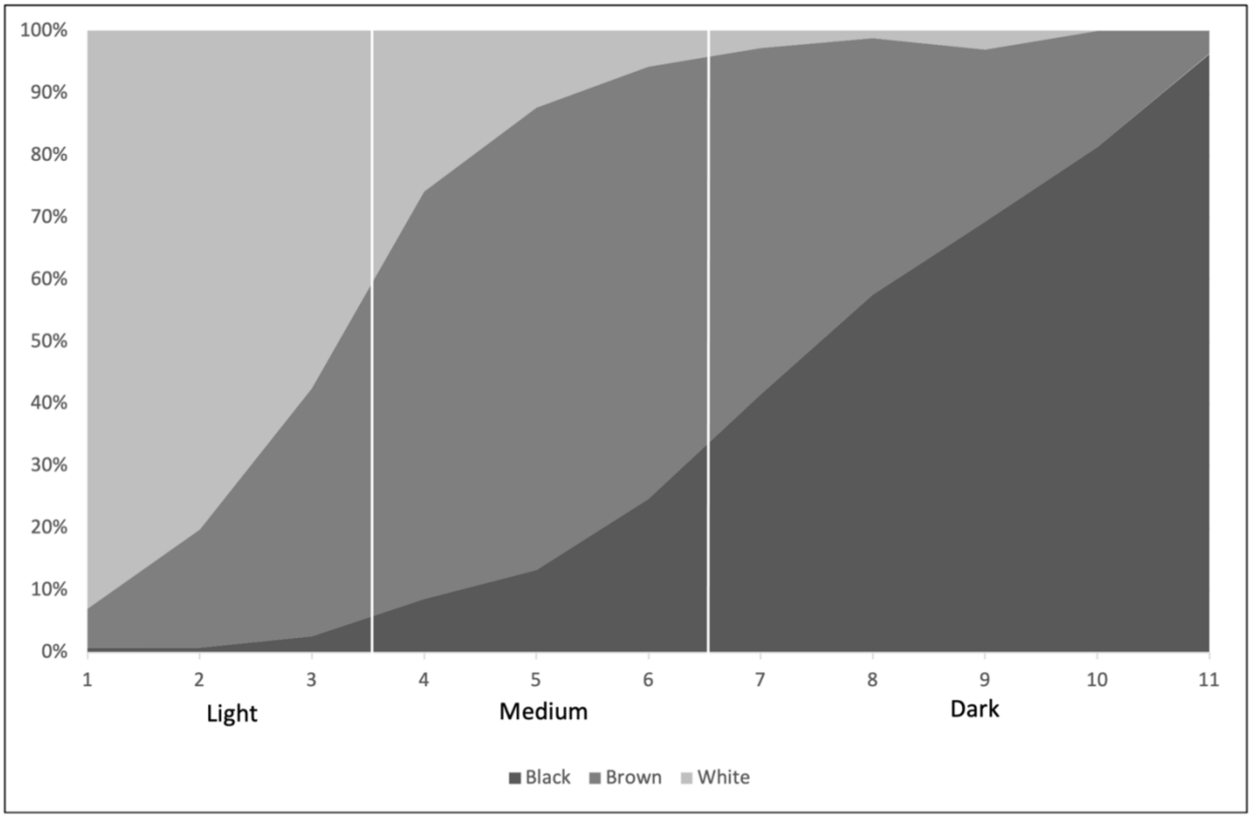

Pooled sample, N = 8804
Source: LAPOP, six survey rounds merged, 2010, 2012, 2014, 2016/17, 2019, 2023

Figure S2 Simulation predicted probabilities of self-identity as white, brown or black for light skin persons (panel A), medium skin persons (panel B) and dark skin persons (panel C)

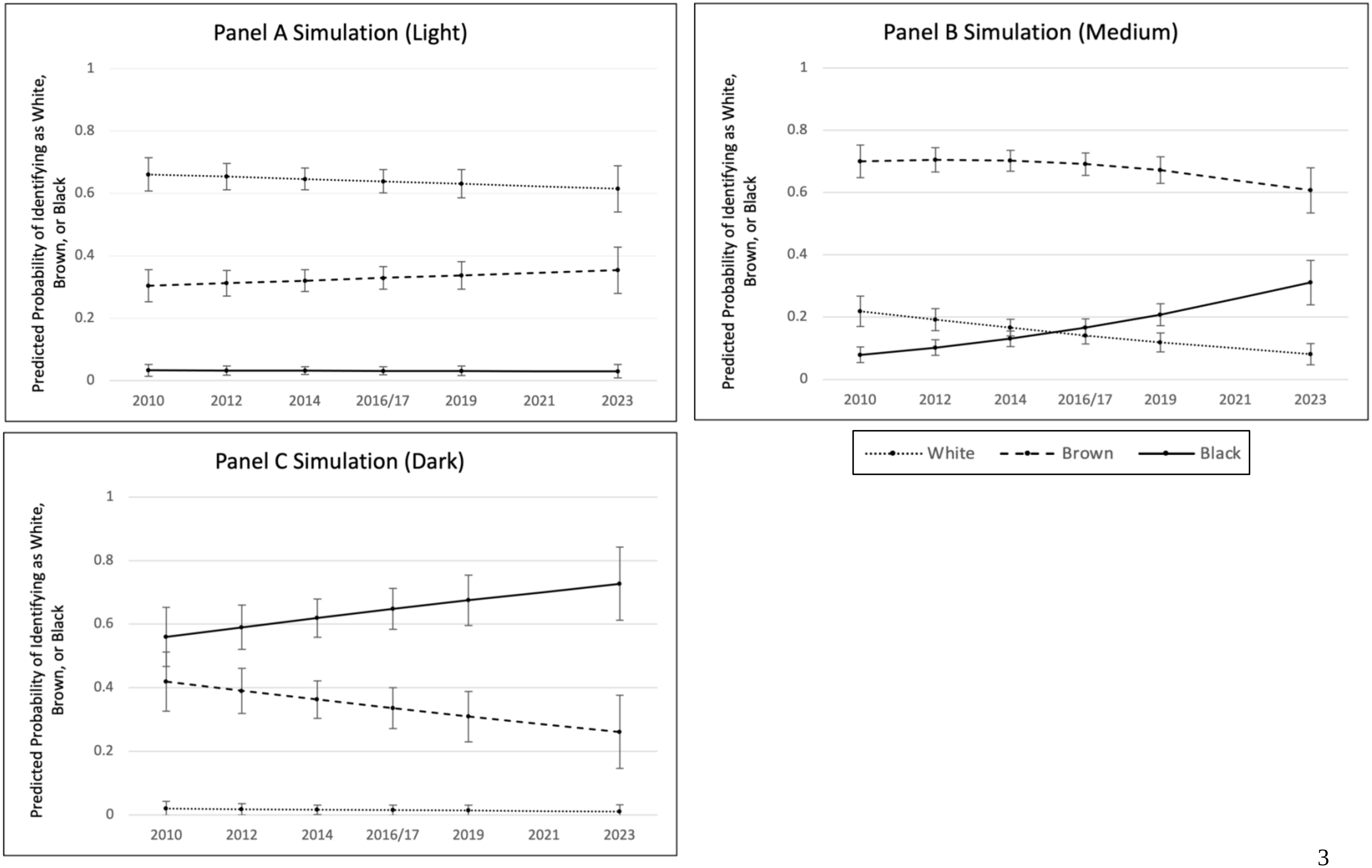

Figure S3 Simulation predicted probabilities of self-identity as white, brown or black, by education level, for light skin persons (panel A), medium skin persons (panel B) and dark skin persons (panel C)

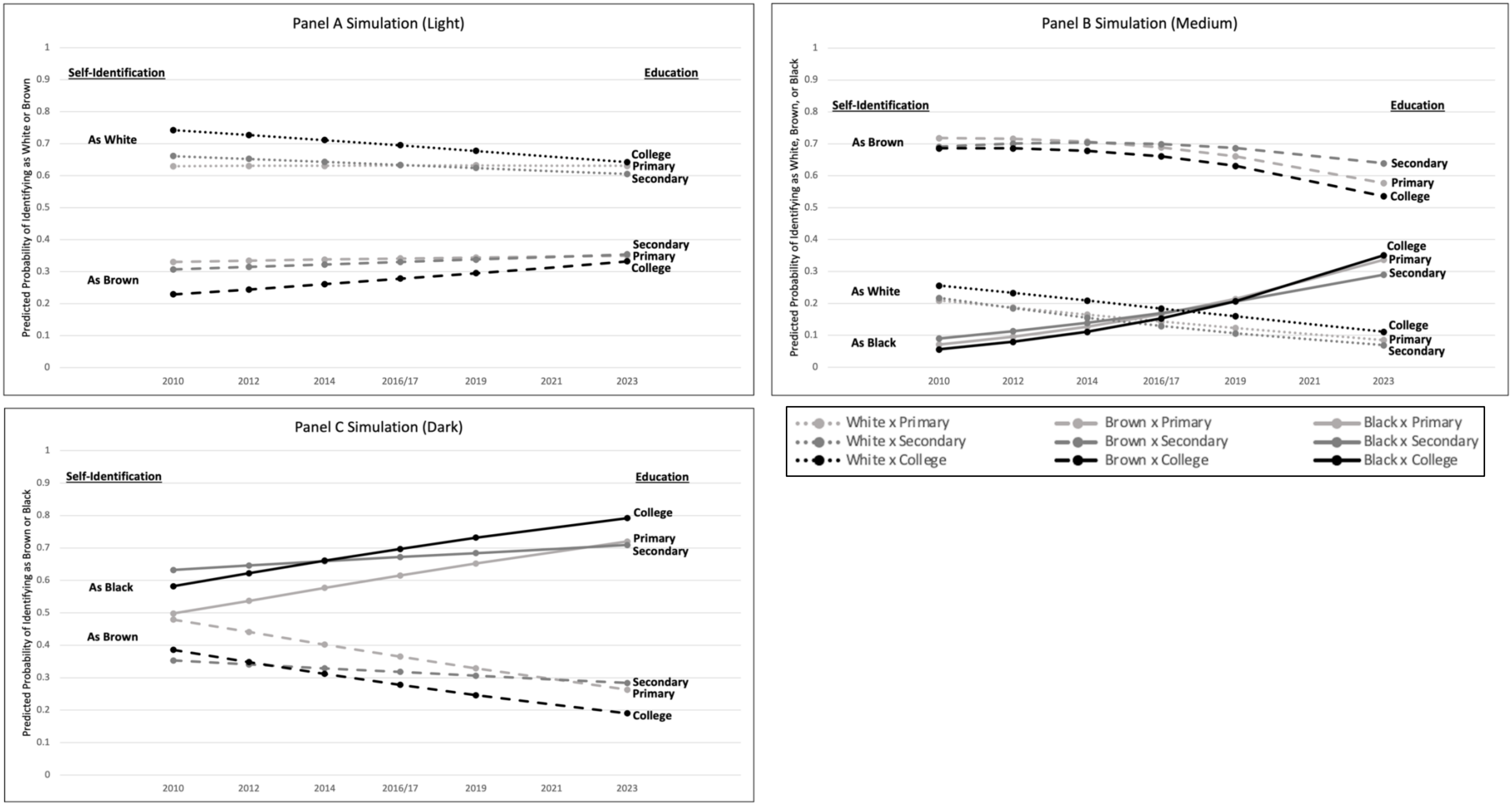

Figure S4 Latin American public opinion project (LAPOP) skin color palette

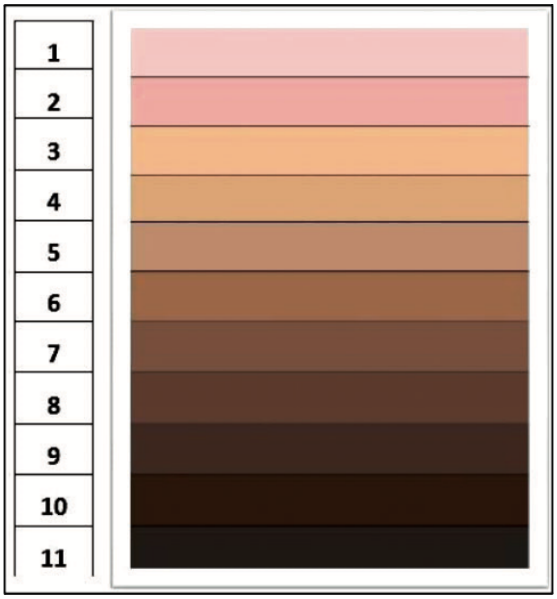

Table S1 Descriptive statistics for variables in regression models

	Mean or %	(SD)
Race		
White	36.1%	
Brown	47.6%	
Black	16.2%	
Respondent skin color		
Light	39.3%	
Medium	46.3%	
Dark	14.2%	
Year		
2010	25.6%	
2012	15.9%	
2014	15.0%	
2016/17	14.4%	
2019	14.1%	
2023	14.8%	
Female	52.0%	
Age	38.6	(15.63)
Highest Education		
Primary	41.1%	
Secondary	45.7%	
College	13.1%	
Wealth	0.01	(1.0)
Urban	87.6%	
Region		
North	9.01%	
Northeast	24.9%	
Central-west	8.85%	
Southeast	40.9%	
South	16.3%	
Interviewer skin color		
Light	43.9%	
Medium	48.6%	
Dark	7.41%	

Pooled sample, $N = 8804$

Source: LAPOP, six survey rounds merged, 2010, 2012, 2014, 2016/17, 2019, 2023

Table S2 Simulation multinomial regressions predicting brown and black versus white racial identity

	Model 1 (without interactions)		Model 2 (with interactions)	
	Brown	Black	Brown	Black
	b	*b*	*b*	*b*
Respondent skin color (reference = light)				
Medium	2.148***	3.036***	1.830***	1.574***
	(0.065)	(0.120)	(0.123)	(0.225)
Dark	3.786***	6.786***	3.841***	6.181***
	(0.239)	(0.257)	(0.367)	(0.399)
Year	0.064***	0.242***	0.037	-0.003
	(0.015)	(0.021)	(0.020)	(0.046)
Female	0.113	0.193*	0.113	0.194*
	(0.061)	(0.087)	(0.061)	(0.088)
Age	-0.014***	-0.018***	-0.014***	-0.018***
	(0.002)	(0.003)	(0.002)	(0.003)
Education (reference = primary)				
Secondary	-0.018	0.130	-0.014	0.127
	(0.076)	(0.106)	(0.076)	(0.107)
College	-0.333***	-0.157	-0.333***	-0.175
	(0.104)	(0.151)	(0.104)	(0.152)
Wealth	-0.106**	-0.037	-0.106**	-0.030
	(0.034)	(0.048)	(0.034)	(0.048)
Urban	0.318***	0.499***	0.313***	0.485***
	(0.096)	(0.148)	(0.096)	(0.150)
Region (reference = southeast)				
North	1.241***	0.307	1.242***	0.308
	(0.109)	(0.166)	(0.109)	(0.167)
Northeast	0.965***	0.921***	0.958***	0.905***
	(0.088)	(0.118)	(0.088)	(0.119)
Central-west	0.717***	0.125	0.708***	0.110
	(0.097)	(0.151)	(0.096)	(0.150)
South	-1.229***	-0.794***	-1.230***	-0.801***
	(0.085)	(0.121)	(0.085)	(0.124)
Interviewer skin color (reference = light)				
Medium	-0.020	-0.091	-0.019	-0.094
	(0.064)	(0.091)	(0.064)	(0.092)
Dark	0.042	0.172	0.038	0.189
	(0.138)	(0.177)	(0.140)	(0.181)
Respondent skin color x year interaction				
Medium x year			0.104**	0.396***
			(0.033)	(0.056)
Dark x year			-0.026	0.137
			(0.100)	(0.106)
Pseudo R^2	0.287		0.290	

Pooled sample, $N = 8804$

Beta coefficients are presented. Robust standard errors in parentheses.

* $p < 0.05$, ** $p < 0.01$, *** $p < 0.001$ (two-tailed tests)

Source: LAPOP, six survey rounds merged, 2010, 2012, 2014, 2016/17, 2019, 2023

Table S3 Simulation multinomial regressions predicting brown and black versus white racial identity (3-way interaction with respondent skin color, education, and year)

	Brown		Black	
	b	(SE)	*b*	(SE)
Respondent skin color (reference = light)				
Medium	1.771***	(0.187)	1.141***	(0.341)
Dark	3.787***	(0.514)	5.631***	(0.567)
Year	0.009**	(0.034)	-0.140	(0.80)
Education (reference = primary)				
Secondary	-0.151	(0.175)	-0.479	(0.399)
College	-0.606**	(0.237)	-0.645	(0.546)
Respondent skin color x year interaction				
Medium x year	0.103	(0.054)	0.547***	(0.094)
Dark x year	-0.052	(0.151)	0.258	(0.164)
Education x year interaction				
Secondary	0.029	(0.045)	0.195*	(0.102)
College	0.076	(0.057)	0.144	(0.143)
Respondent skin color x education interaction				
Medium x Secondary	0.012	(0.265)	0.698	(0.478)
Medium x College	0.374	(0.381)	0.170	(0.729)
Dark x Secondary	0.151	(0.788)	1.130	(0.852)
Dark x College	-0.010	(1.089)	0.393	(1.145)
Respondent skin color x education x year interaction				
Medium x Secondary	0.035	(0.074)	-0.217	(0.123)
Medium x College	-0.091	(0.097)	-0.108	(0.177)
Dark x Secondary	0.104	(0.212)	-0.167	(0.226)
Dark x College	-0.048	(0.256)	-0.108	(0.274)
Pseudo R^2			0.291	

Pooled sample, $N = 8804$
Beta coefficients are presented. Robust standard errors in parentheses.
* $p < 0.05$, ** $p < 0.01$, *** $p < 0.001$ (two-tailed tests)
Controls included, but not presented
Source: LAPOP, six survey rounds merged, 2010, 2012, 2014, 2016/17, 2019, 2023

Table S4 Multinomial regressions predicting brown and black versus white racial identity (with 11-point respondent skin color measure)

	Model 1		Model 2	
	Brown	Black	Brown	Black
	b	*b*	*b*	*b*
Respondent skin color	1.072***	1.756***	1.020***	1.722***
	(0.032)	(0.041)	(0.055)	(0.072)
Year	0.161***	0.392***	0.107*	0.365***
	(0.016)	(0.023)	(0.050)	(0.081)
Female	0.149*	0.219*	0.149*	0.219*
	(0.064)	(0.095)	(0.064)	(0.095)
Age	-0.013***	-0.019***	-0.013***	-0.020***
	(0.002)	(0.003)	(0.002)	(0.003)
Education (reference = primary)				
Secondary	0.022	0.219*	0.023	0.220*
	(0.079)	(0.115)	(0.079)	(0.115)
College	-0.228*	0.062	-0.226*	0.062
	(0.109)	(0.167)	(0.109)	(0.167)
Wealth	-0.043	0.057	-0.044	0.056
	(0.036)	(0.052)	(0.036)	(0.052)
Urban	0.315**	0.458**	0.313**	0.455**
	(0.100)	(0.158)	(0.100)	(0.158)
Region (reference = southeast)				
North	0.849***	-0.098	0.848***	-0.100
	(0.108)	(0.160)	(0.108)	(0.160)
Northeast	0.886***	0.829***	0.882***	0.825***
	(0.093)	(0.130)	(0.093)	(0.130)
Central-west	0.609***	-0.086	0.605***	-0.087
	(0.100)	(0.162)	(0.100)	(0.162)
South	-1.332***	-0.928***	-1.334***	-0.931***
	(0.089)	(0.138)	(0.089)	(0.138)
Interviewer skin color	-0.018	0.026	-0.177	0.026
	(0.019)	(0.028)	(0.019)	(0.028)
Respondent skin color x year interaction			0.016	0.011
			(0.014)	(0.018)
Pseudo R^2	0.360		0.360	

Pooled sample, $N = 8804$
Beta coefficients are presented. Robust standard errors in parentheses.
* $p < 0.05$, ** $p < 0.01$, *** $p < 0.001$ (two-tailed tests)
Source: LAPOP, six survey rounds merged, 2010, 2012, 2014, 2016/17, 2019, 2023
